# A study of meiomitosis and novel pathways of genomic instability in cutaneous T-cell lymphomas (CTCL)

**DOI:** 10.18632/oncotarget.26479

**Published:** 2018-12-28

**Authors:** Matthew Tsang, Jennifer Gantchev, Elena Netchiporouk, Linda Moreau, Feras M. Ghazawi, Steven Glassman, Denis Sasseville, Ivan V. Litvinov

**Affiliations:** ^1^ Division of Dermatology, University of Ottawa, Ottawa, Ontario K1H 8L6, Canada; ^2^ Division of Dermatology, McGill University, Montréal, Québec H4A 3J1, Canada

**Keywords:** genomic instability, meiomitosis, cutaneous T-cell lymphomas, LINE-1, DNA double strand breaks

## Abstract

Genomic instability is a hallmark of cancer and an enabling factor for genetic alterations that drive cancer development and progression. The clashing of mitosis and aberrantly expressed meiosis machineries, which may contribute to genomic instability, has been coined cancer “meiomitosis”. *LINE-1* retrotransposition, a process active in germ cells, acts outside of the meiotic machinery to create DNA double strand breaks (DNA DSBs) and has played an important role in the evolution of the human genome. We have previously demonstrated that in CTCL several cancer testis/meiotic genes are expressed. Furthermore, this cancer exhibits extensive and ongoing chromosomal/microsatellite instability. In this study we analyzed immortalized patient-derived cells and primary CTCL patient samples using RT-PCR, western blotting and confocal microscopy and found that proteins critically involved in meiosis and *LINE-1* retrotransposition are expressed and are associated with chromosomal instability and DNA DSB formation. Using cell cycle synchronization, we show G1/S phase-transition-specific expression of meiosis proteins. Using the Alu retrotransposition assay, we demonstrate the functional activity of *LINE-1* retrotransposon in CTCL. Histone acetyltransferase inhibition results in downregulation of the ectopic germ cell programs and concomitant decrease in DNA DSBs foci formation. Notably, *LINE-1* and meiosis genes were expressed across a panel of other solid tumor cell lines. Taken together, our results indicate that malignant cells in culture undergo “cancer meiomitosis” rather than the classic mitosis division. The ectopic expression of meiosis genes and reactivation of *LINE-1* may be contributing to genomic instability and represent novel targets for immunotherapy in this and other cancers.

## INTRODUCTION

Genomic instability (including chromosomal instability) is considered a hallmark of cancer and has been established as a common driving force behind cancer development and progression [1]. It is understood that the genomic instability in hereditary cancers is linked directly to mutations in DNA repair genes [2]. However, mutations of DNA repair genes are uncommon in sporadic cancers, suggesting that other mechanisms exist to induce/drive genomic instability in these malignancies.

Of particular interest in the context of genomic instability is retrotransposon reactivation. Retrotransposons comprise roughly 40% of the mammalian genome and have played an important role in evolution through their ability to accumulate and autonomously “jump” within the genome [3]. The largest, and the only currently active group of mobile DNA are the *LINE-1* retrotransposons, which constitute ~17% of our genome [4]. *LINE-1* encodes two proteins *ORF1p* and *ORF2p*, which function together to insert mutations into the genome via “copying and pasting” mechanism of its own sequence, or other non-autonomous retrotransposon sequences that fully depend on *LINE-1* machinery to mobilize [5]. For example, *ALU* transposable element is not able to “jump” unless retrotransposed by active *LINE-1* enzyme machinery. When active, *LINE-1* and other retrotransposons can jump and result in deleterious effects by reshuffling the genome and altering gene expression [6]. *LINE-1* can also directly disrupt genes as a result of retrotransposition. Thus, *LINE-1* expression is normally suppressed by DNA methylation to maintain genomic stability in somatic cells [7]. However, this silencing program is lifted in germ cells during epigenetic reprogramming [8], and so retrotransposon suppressors, such as *PIWI* family proteins [9] and *GTSF1* [10] must be activated in order to mitigate genomic mutations/damage by retrotransposons. Notably, *LINE-1* has been shown to be expressed in a number of cancers, likely due to a hypomethylated state of their DNA [5] and in some cases is associated with poor disease prognosis [11].

The other critical mechanism that could promote genomic instability involves ectopic reactivation of expression of germ cell proteins by cancer cells that could drive cancer meiomitosis, a recently coined term describing the clashing of mitosis and meiosis machineries during the cell cycle [12, 13]. Hundreds of proteins specifically expressed by germ cells and cancer cells have been identified, and have been termed Cancer Testis (CT) antigens [14]. Although several CT antigens have been shown to have diagnostic and prognostic value [15], their functions in cancer cells have not been well studied [12]. Of particular interest for oncogenesis are the subset of CT genes that normally mediate the meiotic program and thus possess chromosome modulating potential [16]. A number of meiosis-specific CT genes including, but not limited to *SPO11* [12]*, STRA8* [17]*, DMC1* [18]*, REC8, STAG3* [18]*, SGO2* [12]*, SYCP1/2/3* [18] and *HORMAD1* [12] have been shown to be expressed in various solid and hematological cancers as well as in different cancer cell lines. Due to space limitation we summarize the function of these genes in the Supplementary Appendix and in Supplementary Figure 1 of this manuscript. It has been postulated that this clashing of meiotic and mitotic pathways (i.e., cancer meiomitosis) could give rise to chromosomal instability in dividing cancer cells [13]. Specifically, it has been hypothesized that proteins involved in crossing over, meiotic DNA double strand breaks (DSB) formation and repair, may promote genomic rearrangements [19], while proteins involved in chromosomal cohesion could promote polyploidy [20]; however, no studies have yet been performed to mechanistically verify these statements.

According to the Leukemia & Lymphoma Society, lymphomas are one of the most common malignancies, where ~790,000 people are either living with/or in remission from a lymphoma in the United States alone. The majority of patients (~75%) are diagnosed with non-Hodgkin's Lymphomas. Cutaneous T-Cell Lymphoma (CTCL) is the most common lymphoma of the skin. CTCL is a heterogeneous group of Non-Hodgkin lymphoproliferative disorders characterized by localization of neoplastic T lymphocytes to the skin. Mycosis Fungoides (MF), its leukemic form, Sézary Syndrome (SS) and primary cutaneous anaplastic large cell lymphoma (cALCL) are the most common variants and account for ~80% of all CTCL [21, 22]. The molecular pathogenesis of this cancer (e.g. resistance to apoptosis, presence of chromosomal translocations, upregulation of *BCL-2*, increased *STAT3/STAT5* signaling, etc.) is believed to be similar to other T cell lymphomas [23]. The malignant T cells in cutaneous lymphomas are expressing *Cutaneous Lymphocyte-associated Antigen (CLA)* and, as a result, are homing to the skin, where they are easily accessible for diagnosis and clinical follow up. In other lymphomas, malignant T cells express different homing markers that target them to lungs, gastrointestinal track, etc. (e.g., *VLA1* targets T cells to lungs, while *CCR9* and *Integrin ɑ_4_β_7_* target T cells to intestines). Hence, CTCL represents an intriguing model to study lymphomagenesis.

MF and SS are the most commonly studied variants of CTCL and account for more than 50% of all cases. They differ significantly from cALCL, which is categorized as part of the CD30^+^ lymphoproliferative disorders. In early stages, MF presents with localized erythematous patches and plaques mainly on the trunk. While CTCL presenting at an early stage typically follows an indolent course in a majority of patients, a minority of patients progress to advanced stages [24].

SS is an aggressive form of CTCL that is characterized by a triad of erythroderma, lymphadenopathy and detection of malignant T cells with cerebriform nuclei on a peripheral blood smear [25]. SS typically arises de novo and evolves in a short time period, although some patients may have a prodrome of pruritus, erythema and nonspecific dermatitis [26]. SS patients often have a median disease survival of only 2-4 years [27]. Sézary patients often have dismal quality of life and are constantly suffering from severe itch, recurrent infections and shivering due to ongoing excessive fluid loss in addition to other common leukemia symptoms.

We have previously shown using high throughput screening that CT antigens normally essential for meiosis (*SPO11, REC8, SYCP1*) and a retrotransposon suppression gene (*GTSF1*) together are overexpressed in CTCL lesional skin, when compared to benign inflammatory dermatoses [21, 28, 29]. In this work, we explore through characterization and mechanistic functional experimental studies novel pathways of genomic instability involving meiosis genes and retrotransposons in CTCL using it as a model for other human cancers.

## RESULTS

### Evidence of chromosomal instability in CTCL

**Figure 1:**
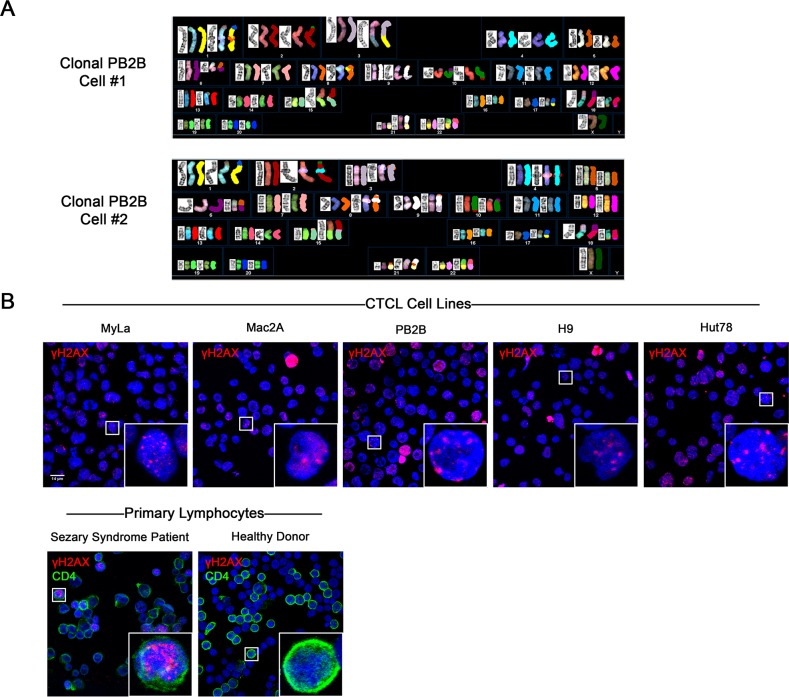
Evidence of genomic instability in CTCL via spectral karyotyping and immunofluorescence staining of DNA double strand breaks (DSBs) **(A)** G banding and Spectral Karyotyping of CTCL cell lines showed extensive chromosomal abnormalities which varied between clonal cells and between cell lines. In this figure, results from two clonal cells of the PB2B cell line are shown. Data for other MF/SS cell lines is presented in Supplementary Figure 2. **(B)** Immunofluorescence expression of *γH2AX*, a marker of DNA DSBs, in CTCL cell lines, and primary lymphocytes isolated from the whole blood of a Sézary Syndrome patient and a healthy donor. Primary lymphocytes were double stained with *CD4* to identify helper T cells. Punctate foci of *γH2AX* were observed in the nucleus of malignant but not healthy lymphocytes. Pan-nuclear *γH2AX* is indicative of apoptotic cells.

Chromosomal and microsatellite instability has previously been extensively documented in MF and SS variants of CTCL [30–33]. We have observed that classic patient-derived CTCL cell lines exhibit chromosomal abnormalities consistent with ongoing chromosomal instability, as detailed in our recent report [34]. G-banding and spectral karyotyping analysis showed that cell lines representative of MF (PB2B, Mac2A, MyLa, HH) and SS (H9, Hut78, SeAx, SZ4 and Sez4) possess unique, extensive, and seemingly random chromosomal abnormalities that vary between clonal cells within the same cell line (PB2B cells shown in Figure 1A, all cell lines in Supplementary Figure 2) [34]. Since DNA double strand breaks are necessary for chromosomal rearrangements to occur, and unrepaired DNA DSBs are associated with genomic instability [35], we also performed immunofluorescence to detect *γH2AX*, a marker of DNA DSBs [36], in various CTCL cell lines, and in lymphocytes derived from an SS patient and a healthy donor (Figure 1B). In all tested CTCL cell lines, multiple punctate foci of *γH2AX* positivity were commonly detected in the nucleus using confocal microscopy. Similarly, multiple foci of *γH2AX* positivity were observed in the nuclei of CD4^+^ lymphocytes from SS patients. In contrast, punctate nuclear foci of *γH2AX* were not detected in stimulated proliferating lymphocytes from a healthy donor, suggesting that the presence of DNA DSBs was a phenomenon unique to malignant T-cells. Pan-nuclear expression of *γH2AX*, indicative of apoptotic cells [37], was observed in occasional lymphocytes in all cell lines and primary samples tested.

### Ectopic expression of germ cell proteins and LINE-1 across CTCL cell lines

**Figure 2:**
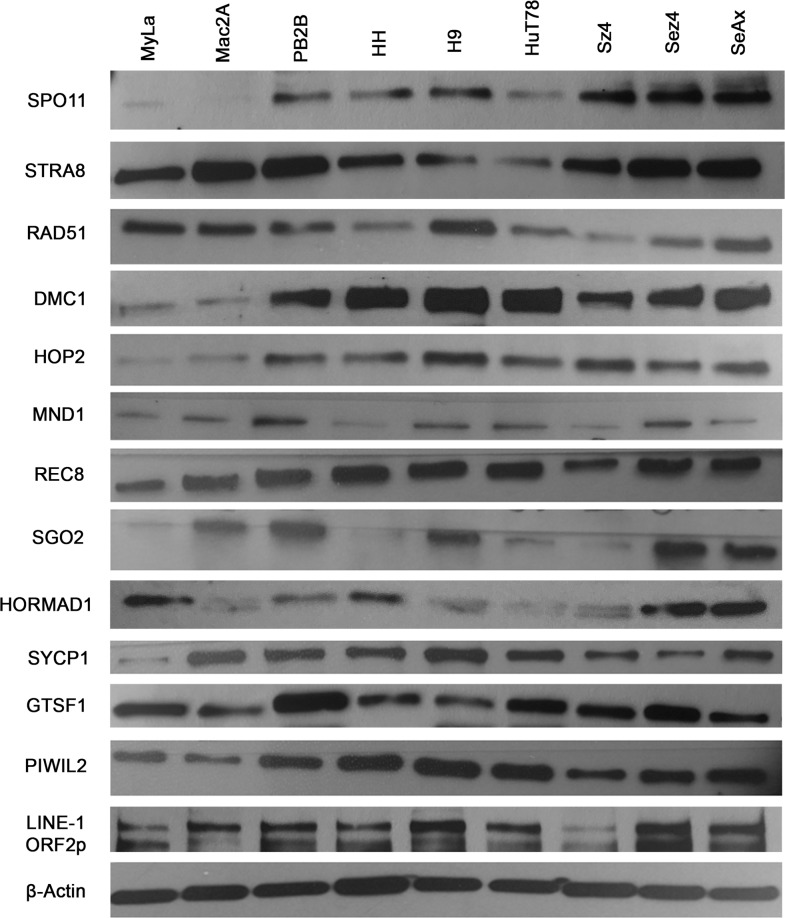
Ectopic expression of meiosis/germ cell genes in CTCL cell lines representative of mycosis fungoides and Sézary syndrome Protein lysates from nine CTCL cell lines (MyLa, Mac2A, PB2B, HH representing Mycosis Fungoides and H9, Hut78, SZ4, Sez4 and SeAx cells representing Sézary Syndrome) were assessed by western blot for ectopic expression of *SPO11, STRA8, RAD51, DMC1, HOP2, MND1, REC8, SGO2, HORMAD1, SYCP1, GTSF1, PIWIL2* and *LINE-1 ORF2p*. *β-Actin* was used a loading control.

**Figure 3:**
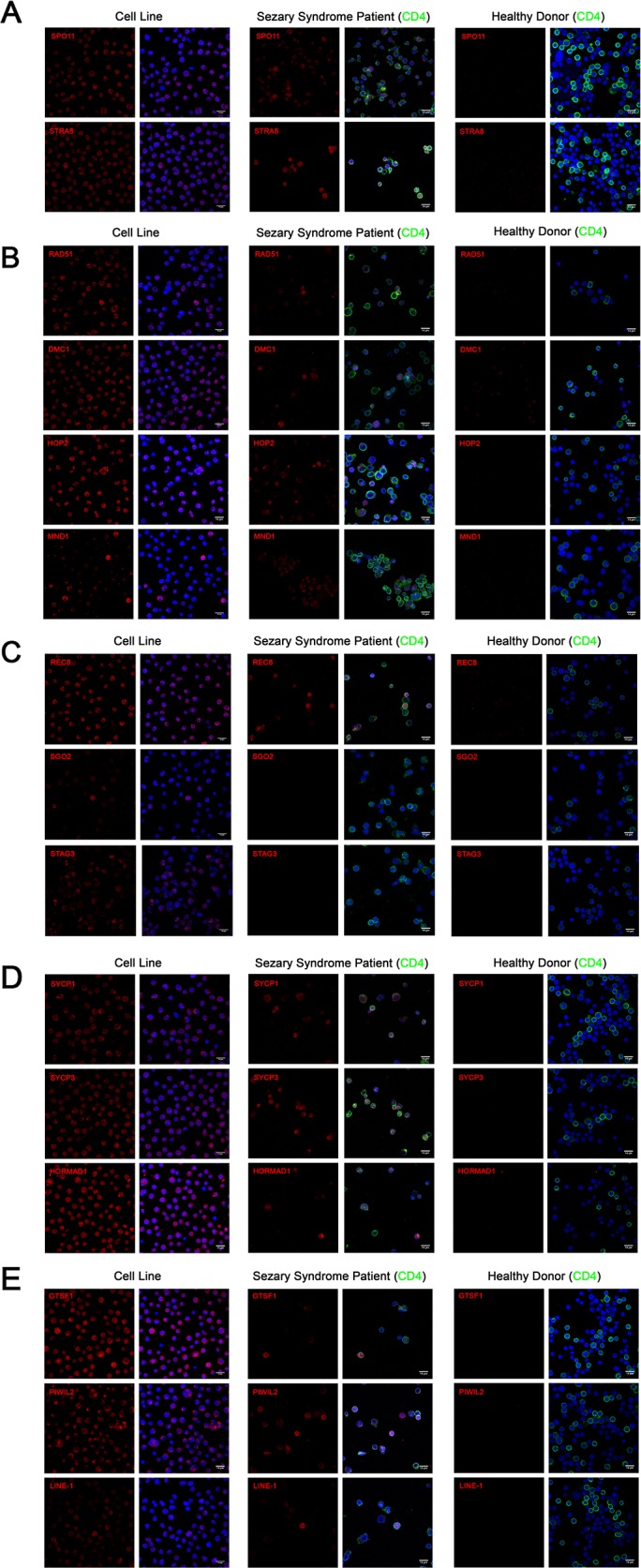
Immunofluorescence of **(A)** meiosis initiation **(B)** homologous recombination **(C)** meiotic cohesin **(D)** meiotic synapsis **(E)** retrotransposon-associated proteins in CTCL cell lines, Sézary Syndrome patient-derived lymphocytes and healthy donor-derived stimulated proliferating lymphocytes. In CTCL cell lines, predominantly nuclear localization was observed for *SPO11, STRA8, RAD51, DMC1, HOP2, REC8, STAG3* and *LINE-1* proteins. Combined nuclear and cytoplasmic staining was observed for *SYCP1, SYCP3, HORMAD1, GTSF1* and *PIWIL2*. Punctate staining was observed for *MND1* and *SGO2*. Heterogeneous expression of these proteins was observed in *CD4^+^* lymphocytes derived from Sézary Syndrome patients (representative images are shown). Expression these proteins could not be detected in lymphocytes from a healthy donor. Scale bar = 14μm.

To screen for the expression of various germ cell proteins that could contribute to genomic instability, we performed western blot analysis of proteins associated with meiosis and *LINE-1* retrotransposition across 9 CTCL cell lines (Figure 2). Proteins with known roles in meiosis initiation (*SPO11, STRA8*), homologous recombination (*RAD51, DMC1, HOP2, MND1*), sister chromatid cohesion (*REC8, SGO2*), synapsis (*SYCP1)* and retrotransposon suppression (*GTSF1, PIWIL2*) were evaluated, along with the *LINE-1 ORF2p* protein. Expression of these proteins in the panel of these cancer cells was also detected via RT-PCR (Supplementary Figure 3). Importantly, as documented in Figure 3A-3E (right panel) using immunofluorescence, proliferating stimulated T lymphocytes isolated from healthy subjects did not express the aforementioned genes.

### Immunofluorescence of germ cell and LINE-1 proteins in CTCL cell line, Sézary syndrome patient-derived, and healthy donor-derived lymphocytes

To corroborate our western blot results from immortalized CTCL cell lines, immunofluorescence staining using confocal microcopy was performed on PB2B cells, a cell line which strongly expressed our panel of germ cell proteins, as well as on primary lymphocytes expanded from the peripheral blood of a Sézary Syndrome patient and those from a healthy donor. Stained cells were analyzed with confocal microscopy in order to visualize the cellular localization of germ cell proteins (Meiosis Initiation: *SPO11, STRA8*; Homologous Recombination: *HOP2*, *MND1, DMC1, RAD51*; Meiotic Cohesins: *REC8, SGO2, STAG3*; Meiotic Synapsis: *SYCP1, SYCP3, HORMAD1*; Retrotransposon-associated genes: *GTSF-1, PIWIL2, LINE-1 ORF2p*). Staining for each protein resulted in unique expression patterns that were either predominantly nuclear, combined nuclear and cytoplasmic, or punctate (Figure 3A-3E, left panel). In immortalized CTCL cells, *SPO11, STRA8, HOP2, DMC1, RAD51, REC8, STAG3* and *LINE-1* exhibited specific nuclear expression, which generally spared the nucleoli, while *SYCP1, SYCP3, HORMAD1*, *GTSF1* and *PIWIL2* were expressed diffusely in both the nucleus and cytoplasm. Punctate staining was observed for *MND1*, where nuclear and perinuclear foci were observed in addition to a subset of cells displaying strong diffuse nuclear and cytoplasmic staining, and *SGO2*, where foci localized to the centromeres of mitotic cells (confirmed by double staining with *CENPA* antibody, data not shown).

In lymphocytes expanded from the peripheral blood of a clinically advanced SS patient, expression of most germ cell proteins we assessed could be detected in *CD4^+^* malignant T cells in a similar staining pattern to that we observed in the immortalized cells (Figure 3A-3E, middle panel). Consistent with the mixed population of healthy and malignant T cells in the peripheral blood of SS patients, we observed heterogeneity in the expression of these proteins, which exhibited cell-to-cell variation in staining intensity. In contrast, none of the germ cell proteins we assessed could be detected by immunofluorescence in stimulated dividing lymphocytes derived from a healthy donor (Figure 3A-3E, right panel).

### Temporal regulation of meiosis protein expression

**Figure 4:**
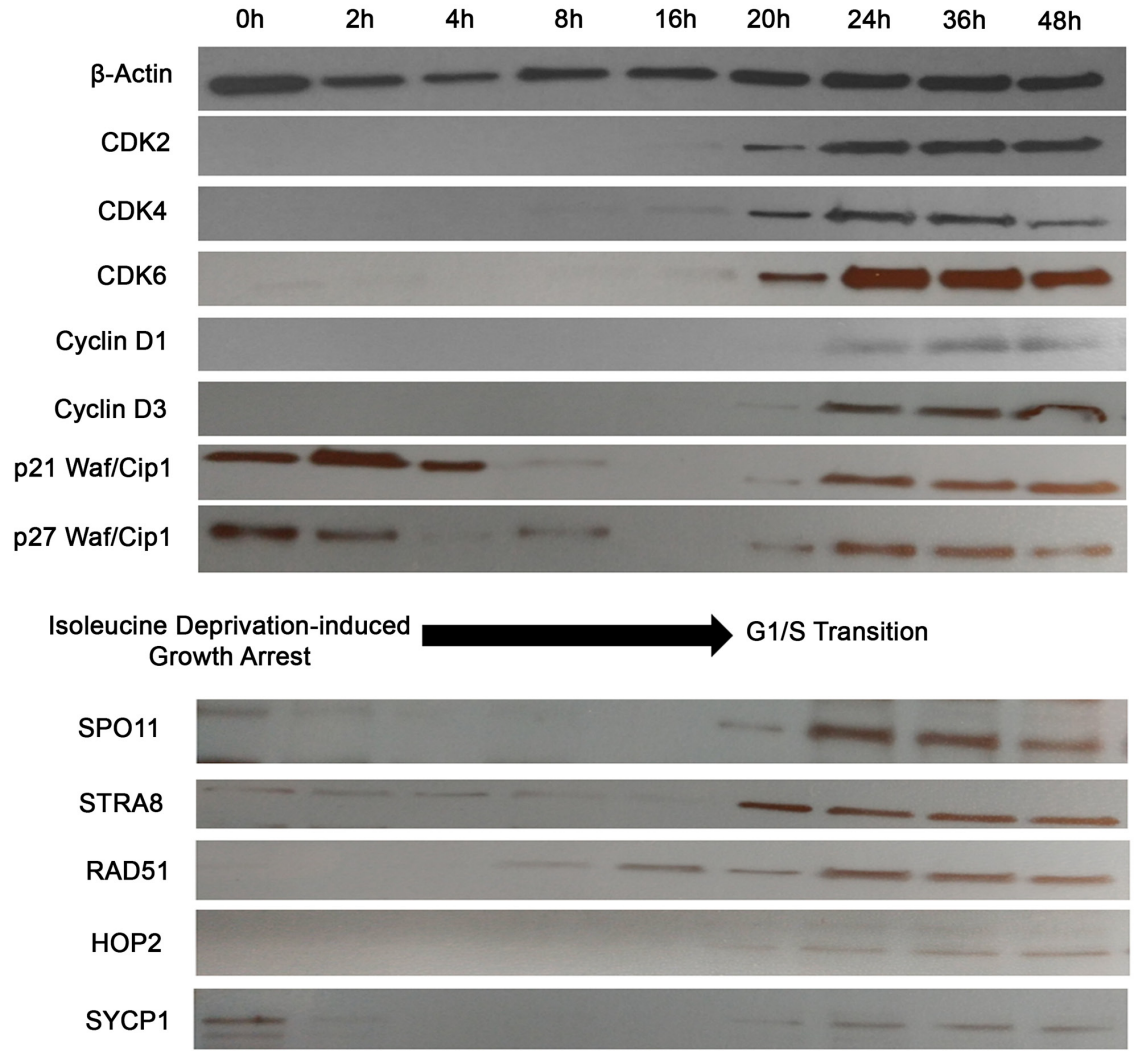
Activation of meiosis proteins during G1/S phase transition in synchronized CTCL cells PB2B cells were incubated in isoleucine-deficient media for 24h to induce growth arrest, then transferred back to proliferation media and harvested for western blot analysis after incubation for 0, 2, 4, 8, 16, 20, 24, 36 and 48 hours. Cell cycle markers including *CDK2, Cyclin D3* and *p21* were analyzed to correlate time points to phases of the cell cycle. *SPO11, STRA8, RAD51, HOP2* and *SYCP1* were most strongly activated during the time of G1/S phase transition at 20-24 hours.

The above analyses provide a static picture of expression of these ectopically expressed proteins. The question then arises whether the expression of these CTA/germ cell proteins is an epiphenomenon, where these proteins get aberrantly expressed in cancer cells or whether these genes are tightly regulated with respect to the stage of cell cycle throughout mitosis (or so called, cancer meiomitosis). To answer this question, we synchronized growth arrest (i.e., a G0 phase) in PB2B cells using RPMI media deficient in isoleucine, and protein expression was evaluated at 0, 2, 4, 8, 16, 20, 24, 36 and 48 hours after cells were transferred back into proliferation media (Figure 4). We used expression of known cell cycle proteins to assess whether cancer cells tightly regulate the expression of these genes throughout the cell cycle similarly to healthy germ cells. Specifically, *CDK2/4/6* are normally expressed during the G1 to S phase transition [38] and were induced between 16 and 20 hours in this experiment. *Cyclins D1* and *D3,* which regulate entry into S phase by complexing with *CDK4/6* [39], were induced between 20 and 24 hours (Figure 4). The CDK inhibitors *P21 Waf/Cip1* and *p27 Waf/Cip1* are negative regulators of the cell cycle [40] and were expressed from 0-8 hours, and then re-expressed from 20-48 hours. Taken together, this data suggested that the G1-S phase transition occurred at approximately 20 hours after the cell cycle was resumed in PB2B cells. Corresponding to this, *SPO11, STRA8, HOP2* and *SYCP1* were induced at 20 hours, while *RAD51* was initially detected at 8-hour time point. Perhaps related to its physiological role as the initial switch from mitosis to meiosis, STRA8 was expressed abruptly and reached the highest level of expression at 20 hours before *SPO11, RAD51, HOP2, SYCP1* were induced to their maximal expression. Hence, it appears that these proteins are not randomly or ubiquitously expressed in the cells, but are, in fact, tightly regulated in relation to the stage of the cell cycle, as might be expected considering the function of these genes.

### Functional assessment of LINE-1 retrotransposition in CTCL cells

**Figure 5:**
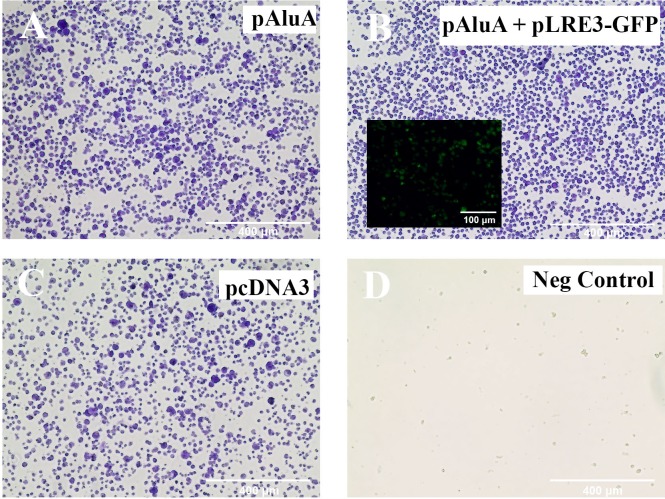
*LINE-1* machinery is functionally active in PB2B cells A previously described *in vivo* assay [42] was performed to test the ability of endogenously expressed *LINE-1* to provide the necessary protein machinery for Alu retrotransposition to occur. PB2B lymphocytes were transfected with **(A)** *pAluA* “reporter” plasmid containing an *Alu* element and a modified mneoI cassette that allows the detection of a retrotransposed *RNA polymerase III* transcribed RNA, **(B)** *pAluA* “reporter” plasmid plus “driver” retrotransposition-competent human *LINE-1* element *LRE3* tagged with *GFP* (inlay) to monitor transfection efficiency **(C)** empty vector control plasmid with the *CMV* promoter and a neomycin-resistance marker **(D)** negative control: plasmid not coding for drug resistance. Transfected cells were grown in suspension and treated with G418 for 6-8 weeks to select for antibiotic resistance conferred in (A) and (B) by retrotransposition events. Cells were subsequently fixed and stained with crystal violet. Images of entire wells (outer panels) and brightfield microscopy are shown (Scale bar = 400 μm).

To determine whether the detected *LINE-1* retrotransposon was functionally active in CTCL cells, we used a recently described Alu retrotransposition assay [41]. This assay is based on the principle that the proteins encoded by *LINE*-1 (*ORF1p* and/or *ORF2p*) act *in trans* to retrotranspose *Short INterspersed Element (SINE)* RNAs such as *Alu* element, which are otherwise unable to retrotranspose. PB2B cells, which represent Mycosis Fungoides, (Figure 5) and H9 cells, which represent Sézary Syndrome, (Supplementary Figure 4) were transfected with an Alu “reporter” plasmid containing an Alu element and a modified *mneoI* cassette that allows the detection of a retrotransposed RNA polymerase III transcribed RNA via G418 resistance. In this assay, cells develop resistance to G418 if *LINE-1* is functional and is able to retrotranspose the Alu element. Our results showed that a number of transfected/electroporated cells survived and were selected after 6-8 weeks of treatment with G418 (Figure 5A, Supplementary Figure 4A), which indicates that the expressed *LINE-1* retrotansposon is fully functional. Similar viability outcome was observed when PB2B or H9 cells were co-transfected or electroporated with the Alu “reporter” and exogenously supplied retrotransposition-competent human *LINE-1* element *LRE3* (Figure 5B, Supplementary Figure 4B). Transfection/electroporation with the antibiotic resistance gene alone (pCDNA3 construct) was performed as an additional positive control, and resulted in cell survival in the presence of G418 (Figure 3C, Supplementary Figure 4C); in contrast, no cells survived, when transfected with an empty control plasmid (Figure 5D, Supplementary Figure 4D).

### Role of histone acetylation in the regulation of ectopic germ cell and LINE-1 protein expression and genomic instability in CTCL

**Figure 6:**
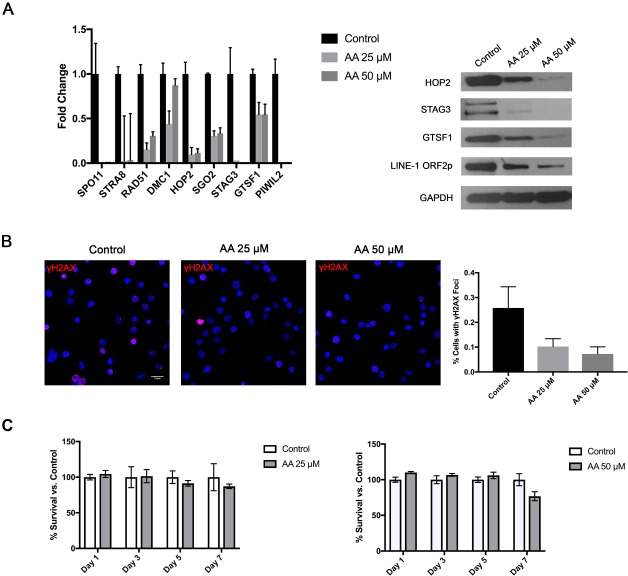
Treatment of CTCL cells with a *Histone Acetyltransferase (HAT)* inhibitor attenuates germ cell program and is associated with reduction in DNA DSBs, but no significant change in cell survival Treatment of PB2B cells with 25 and 50 μM Anacardic Acid (AA) resulted **(A)** in a dose dependent downregulation of the tested meiosis/germ cell gene mRNA expression as tested by RT-PCR (left panel) and corresponding protein expression as tested by a western blot (right panel) as well as **(B)** a significant decrease in DNA double strand break (DSB) formation as visualized using *γH2AX* staining (left panel). Quantification of staining is presented in a graph (right panel), as a mean percentage of cells with distinct *γH2AX* foci (mean ±SD; twelve high powered fields). **(C)** As documented by MTT assay, AA did not significantly affect cell survival at 25 and 50 μM doses.

Recently, epigenetic changes became a significant focus of basic and clinical research in CTCL. A number of previous studies documented methylation/histone acetylation abnormalities in malignant CTCL cells [42, 43]. In fact, two of the commonly used medications for advanced stages of this cancer are *histone deacetylase (HDAC)* inhibitors (Romidepsin and Vorinostat) [44]. Hence, we hypothesized that the widespread ectopic expression of Cancer Testis and *LINE-1* genes/proteins was due to a loss of epigenetic transcriptional repression. To test whether histone acetylation mediates the expression of these genes we treated PB2B cells for 24 hours with *Histone Acetyltransferase (HAT)* inhibitor, Anacardic Acid (AA). As expected, decreasing the baseline rate of histone acetylation in these cells resulted in a corresponding dose-dependent downregulation of the tested germ cell genes mRNA, as documented by RT-PCR (Figure 6A left panel), and proteins as evaluated by Western Blotting (Figure 6A right panel). Consistent with this, we also observed that cells treated with AA exhibited a significant reduction in the percentage of cells with nuclear γH2AX foci as demonstrated by immunofluorescence (Figure 6B; P<0.001, one-way ANOVA, mean of twelve high powered fields per treatment). Notably, as demonstrated by an MTT assay, cell survival was not affected significantly by the AA treatment in these cells (Figure 6C).

### Expression of germ cell and LINE-1 proteins in a panel of other cancer cell lines

**Figure 7:**
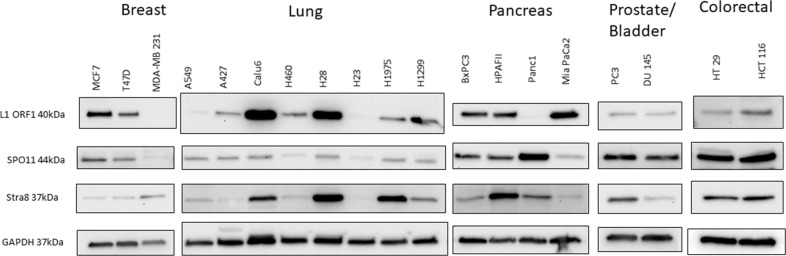
Western blot analysis of protein expression of *LINE-1 ORF1* and early regulatory meiosis genes (*SPO11* and *STRA8*) in a panel of patient-derived breast, lung, pancreas, prostate, bladder and colorectal cancers Expression of the tested meiosis genes is not restrictuted to CTCL, but can also be seen across a panel of other solid tumor cell lines.

Finally, we suspected that the expression and function of these genes and *LINE-1* is not restricted to CTCL. Therefore, we surveyed the expression of meiosis (mRNA and protein levels) and *LINE-1 ORFp1* in breast (MCF7, T47D, MDA-MB 231), lung (A549, A427, Calu6, H460, H28, H23, H1975, H1299) prostate (PC3), bladder (DU145), colorectal (HT29 and HCT116) and pancreatic (BxPC3, HPAFII, Panc1, MiaPaCa2) cancer cells and found that the above described machineries are also expressed across a broad spectrum of patient-derived cell lines representing these malignancies (Figure 7, due to space limitations only Western Blot data for *SPO11, STRA8* and *LINE-1* are shown).

## DISCUSSION

Cells in normal somatic tissues undergo mitosis, while germ cells undergo meiosis. Cancer is not normal and does not follow the rules prescribed for normal cells. In this study we document that CTCL and other cancer cell lines in culture express a number of meiosis/cancer testis genes and using CTCL as a model for cancer, we document that these genes are regulated throughout the cell cycle, are epigenetically controlled and are associated with increased levels of DNA DSB formation. Hence, it appears that immortalized and primary cancer cells obtained from patients in culture undergo abnormal cell division, where the clashing of mitosis and aberrantly expressed meiosis machineries occurs – a process that has recently been named “cancer meiomitosis” [13].

Chromosomal instability has long been associated with progression and poor prognosis of human cancers, including CTCL [45]. This clashing of mitosis and aberrantly expressed meiosis machineries may be contributing to genomic instability in cancer. *LINE-1* retrotransposition is a process active in germ cells which acts outside of the described meiosis machinery to create DNA DSBs [46], generate insertional mutations [6] as well as intra-chromosomal deletions, duplications and translocations [47]. *LINE-1* has been shown to be expressed in several cancers and has been found to be associated with genomic instability and poor prognosis [7].

Our experiments provide novel evidence that ectopically expressed meiosis proteins are associated with genomic instability in CTCL. We have found that these proteins are upregulated during G1-to-S phase transition of the cell cycle, which represents a critical period during which DNA is replicated and is most vulnerable to genome destabilization. This finding reveals that that the expression of these proteins in CTCL is not merely a random epiphenomenon, but can be turned on and off in response to cellular events. With regards to the genome modifying ability of the *LINE-1* retrotransposon, we have found that not only is the *LINE-1* protein expressed in CTCL cells, but that the complete retrotransposition machinery necessary for generating DNA DSBs and intra-chromosomal rearrangements is functional. It is notable that this retrotransposition activity occurs even in the presence of the retrotransposon suppressors *GTSF1* and *PIWIL2* that were found to be highly expressed in all CTCL cell lines and in patients.

Taken in the context with the existing evidence that functional *LINE-1* activity is associated with chromosomal instability in cultured cells [47], this finding provides additional evidence for a novel role of *LINE-1* in CTCL and other human cancers. Moreover, we found that inhibiting histone acetylation in CTCL cells resulted in a dose dependent downregulation of meiosis genes as well as the *LINE-1* protein, which was accompanied by a concomitant reduction in nuclear DNA DSB foci. This finding demonstrates a link between chromosomal instability and the aberrantly expressed germ cell programs and supports the notion that activated “cancer meiomitosis” machinery can promote the formation of DNA DSBs and facilitate chromosomal rearrangements. Further studies are warranted to confirm this relationship in a larger number of patients, and to elucidate the role of specific meiosis genes in disease progression, as the extent of their individual contribution to genomic instability remains unclear. Nevertheless, due to the demonstrated specificity for malignant cells, the meiosis genes we have analyzed represent potential diagnostic/prognostic markers as well as novel targets for immunotherapy.

It is of interest to address the question of why germ cell proteins are widely expressed in CTCL. Indeed, extensive gene expression changes have been reported in MF and SS, in addition to those presently described. However, genomic studies in CTCL have not demonstrated a clear mapping of DNA sequence or chromosomal alterations to these abnormalities [45]. To explain this phenomenon, underlying epigenetic mechanisms including both promoter hypermethylation and hypomethylation are thought to induce the widespread gene expression abnormalities seen in this lymphoma. For instance, hypermethylation of tumor suppressor genes involved in DNA repair, cell cycle, and apoptosis have been characterized in CTCL [48], as has hypermethylation of the *hMLH1* gene, which has been shown to be associated with microsatellite instability in this cancer [31]. A recent study has also found that promoter-specific hypomethylation induces the ectopic expression of the cancer-related genes *PLS3, GATA6* and *TWIST1* in SS [49]. Remarkably, it has been demonstrated that 80% of mice with a knockdown of *DNMT1*, which results in a hypomethylated genome, develop mature T-cell lymphomas [50]. Since *LINE-1* promoter methylation is known to be responsible for its suppression in somatic cells [51], our finding that LINE-1 proteins are expressed highlights the overall hypomethylated state in CTCL cells. It is thus seems likely that a hypomethylated state is responsible for the widespread expression of germ cell proteins in cancer. Our experimental finding that *HAT* inhibition ameliorates this phenomenon adds to the existing evidence that aberrant histone modifications are responsible for atypical and deleterious gene expression in CTCL. Furthermore, the major role of epigenetics in the pathogenesis of CTCL is underscored by the clinical use of histone deacetylase inhibitors Romidepsin and Vorinostat to treat refractory, advanced stage disease [52].

In conclusion, CTCL exhibits chromosomal instability, which may result from expression of normally silenced germ cell programmes. Ectopic expression of meiosis proteins and activation of *LINE-1* retrotransposon machinery represent potential mechanisms through which this type of genomic instability may arise.

## MATERIALS AND METHODS

### Cell culture

MyLa cells represent advanced skin MF, Mac2A/PB2B cells represent advanced skin CD30^+^ MF, while HH cells represent leukemic CD30^+^ MF. SeAx, Sez4/SZ4 and Hut78/H9 cell lines represent true Sézary Syndrome [34]. As detailed in our recent study, Sez4 and SZ4 genetically represent the same cell line. Also, we showed that the same malignant clone caused skin lesions in the patient that gave rise to Mac2A and PB2B cells. Hence, the Mac2A and PB2B cells represent the same malignant tumor, but just at different time points [34]. H9 and Hut78 cells represent the same clinical case/event (H9 is a clonally derived variant from Hut78 cells).

MCF7, T47D, MDA-MB231, A549, A427, Calu6, H460, H28, H23, H1975, H1299, BxPC3, HPAFII, Panc1, MiaPaCa2, PC3, DU145, HT29 and HCT116 cells were purchased from the American Tissue Culture Collection (ATCC) or were obtained from the MUHC-RI Tissue culture core facility and were grown according to manufacturer's recommendations. HH, H9 and Hut78 patient-derived CTCL cell lines were purchased from the ATCC. MyLa, PB2B, Mac2A, SZ4, SeAx, Sez4 were a generous gift from professors K. Kaltoft and N. Ødum (Copenhagen, Denmark) [34]. All cells were grown in 5% CO2, 95% air humidified incubator at 37°C. To block *HAT* activity, cells were treated with 25-50 μM of Anacardic Acid (Sigma-Aldrich, St. Louis, MO). Lysates for western blotting were obtained and quantitated as previously described [34]. Samples from all cell lines were analyzed by G-banding and spectral karyotyping as previously described [34]. MTT assay reagents were obtained from Sigma-Aldrich and were performed as previously described [53].

Primary patient lymphocytes were isolated using Lymphoprep™ Density Gradient Medium (STEMCELL Technologies Inc., Vancouver, BC) as per manufacturer's instructions. All patients were enrolled in an Research Ethics Board (REB)-approved study protocol (The Ottawa Hospital REB study #20150896-01H) with informed consent in accordance with the Declaration of Helsinki. Summary of patient clinical characteristics is presented in Supplementary Table 1. CTCL patients were recruited from the Cutaneous Lymphoma Clinic at The Ottawa Hospital. Peripheral blood was collected in EDTA tubes from Sézary Syndrome patients and a healthy donor at The Ottawa Hospital. Briefly, blood was mixed with an equal volume of PBS, carefully pipetted over Lymphoprep™ Density Gradient Medium and centrifuged for 30 minutes at 1000xg. The resultant layer of Peripheral Blood Mononuclear Cells (PBMCs) was then isolated, washed twice with PBS, and grown in RPMI medium supplemented with 10% FBS and 1.5% PHA (phytohaemagglutinin, Sigma-Aldrich, St. Louis, MO) for 24 hours in a 37°C, 5% CO_2_ tissue-culture incubator. Lymphocytes, which remained in suspension, were subsequently transferred to new flasks containing RPMI medium supplemented with 10% FBS and 20 ng/ml human recombinant IL-2 and were incubated at 37°C for 5-7 days before being analyzed.

### Immunocytochemistry

Indirect immunofluorescence was performed by adhering and staining lymphocytes onto 8-well chamber slides (Thermo Fisher) coated with Poly-L-Lysine (Sigma-Aldrich) as previously described in our protocol [54]. A Zeiss LSM 510 confocal microscope (Zeiss, Oberkochen, Germany) was used for all cell imaging. Multiple planes were acquired using the Z-stack feature, and processed using ImageJ 1.6 software (National Institutes of Health, Bethesda, MD, USA). The following primary antibodies and their dilutions were used: *γH2AX* (Abcam, 1:5000), CD4 (Santa Cruz, Dallas, TX; 1:50), *SPO11* (Bioss, Woburn, MA; 1:50), *STRA8* (Novus Biologicals, Littleton, CO; 1:50), *HOP2* (ProteinTech, Chicago, IL; 1:50), *MND1* (ProteinTech; 1:50), *DMC1* (Santa Cruz; 1:50), *RAD51* (Thermo Fisher; 1:100), *REC8* (ProteinTech; 1:100), *SGO2* (Bethyl Laboratories, Montgomery, TX; 1:50), *STAG3* (Santa Cruz; 1:50), *SYCP1* (Novus Biologicals; 1:100), *SYCP3* (Santa Cruz; 1:50), *HORMAD1* (ProteinTech; 1:100), *LINE-1* (Santa Cruz; 1:50), *GTSF1* (Abnova; 1:100), *PIWIL2* (Santa Cruz; 1:50). The following secondary antibodies and their dilutions were used: Donkey Anti-Mouse IgG H&L Alexa Fluor® 488 (Abcam, 1:250), Donkey Anti-Rabbit IgG H&L Alexa Fluor® 555(Abcam, 1:250), Donkey Anti-Goat IgG H&L Alexa Fluor® 647 (Abcam, 1:250).

### Western blotting

Western blotting was performed as described previously [55]. *SPO11* (Abcam), *STRA8* (Novus Biologicals), *HOP2* (ProteinTech), *MND1* (Santa Cruz), *DMC1* (Santa Cruz), *REC8* (Proteintech), *SGO2* (Bethyl Laboratories), *STAG3* (Novus Biologicals), *SYCP1* (Santa Cruz), *HORMAD1* (ProteinTech), *GTSF1* (Abnova), *PIWIL2* (Santa Cruz) and *LINE-1 ORF2p* (Santa Cruz), *Beta-Actin* (Santa Cruz) and *GAPDH* (Abcam) antibodies were purchased from their respective vendors. *CDK2, CDK4, CDK6, Cyclin D1, Cyclin D3, P21 Waf1/Cip1* and *P27 Waf1/Cip1* antibodies were purchased from Cell Signaling Technology (Danvers, MA) as part of Cell Cycle Regulation Sampler kit (Catalog #9932). Chemiluminescent detection reagents (ECL) were purchased from Amersham Biosciences (Piscataway, NJ).

### Quantitative real-time reverse transcription-PCR gene expression analysis

Gene expression was tested via RT-PCR in CTCL cell lines and in patients. Primer pair sequences for tested genes are listed in Supplementary Table 2. RT-PCR was performed utilizing the obtained cDNA and iScript RT-PCR mix (Bio-Rad, Mississauga, Ontario) on Bio-Rad iCycler as previously described [28, 29, 56]. For every gene analyzed the highest expression value in our samples was set as 1 fold of expression similarly to the protocol in our previous studies [28, 29, 56].

### Cell cycle synchronization

Lymphocytes growing in RPMI proliferation medium containing 10% FBS were washed with PBS and transferred at a concentration of 1×10^7^ cells/mL to new 150cm^2^ flasks containing a custom formulation of RPMI media deficient in Isoleucine (MyBioSource, San Diego, CA). Cells were incubated in isoleucine-deficient medium for 24 hours at 37°C, to induce synchronized growth arrest. To reinitiate the cell cycle, lymphocytes were transferred back to proliferation medium, plated on 100 mm x 15 mm culture dishes and incubated at 37°C for 0, 2, 4, 8, 16, 20, 24, 36 and 48 hours before being harvested for protein analysis. After 48 hours in culture the cells became more asynchronous and expression results were more challenging to interpret.

### LINE-1 functional assay

The following plasmids for human LINE-1 analysis were kindly provided by Dr. John Moran (University of Michigan, USA) and were previously described [42]; *pAluA, pLRE3-GFP*, and *pcDNA3*. *pAluA* is a *puc18* based plasmid that contains the *Alu* sequence recovered from *NF1* gene. It contains a *7SL* promoter and a self-splicing intron within the neomycin indicator cassette with 44 adenine nucleotides at the 3′ end. *pLRE3-GFP* is a *pCEP4* based plasmid that contains the active human L1 (*LRE3*) with a green fluorescent protein (*GFP*) retrotransposition indicator cassette. *pcDNA3* is an empty vector control with the *CMV* promoter driving a neomycin-resistance marker. Plasmid DNA for transfection was isolated from bacteria using Qiagen Midiprep Plasmid DNA kit (Qiagen) according to the manufacturer's instructions. PB2B (Mycosis Fungoides) or H9 (Sézary Syndrome) cells were transferred to pre-warmed RPMI-1640 media supplemented with 10% FBS without penicillin and streptomycin and incubated for 24 hours. For transfection, 5×10^5^ cells were isolated for each of the 3 treatment conditions, as well as a negative control, and were transfected using Lipofectamine 2000 (Invitrogen) with 2μg of plasmid DNA following the manufacturer's instructions. After 24 hours in Lipofectamine, cells were centrifuged and the medium was changed to a complete RPMI media with 100 U/mL penicillin-streptomycin (Invitrogen). For electroporation, 5×10^6^ cells were re-suspended in 100μL of Human T Cell Nucleofector^TM^ Solution (Lonza, Germany) for each condition. A total of 2.5μg of each plasmid was added to the mixture, transferred into an Amaxa-certified cuvette and placed into the Nucleofector^TM^ 2b Device (Amaxa Biosystems). The X-001 program was used for electroporative nucleofection [57]. Immediately following nucleofection, cells were transferred into 12 well culture plates containing 1mL of RPMI-1640 supplemented with 10% FBS, and 100 U/mL penicillin-streptomycin. 48 hours following the transfection of either H9 or PB2B lymphocytes, cells were pelleted and re-suspended in complete media containing 400μg/mL G418 to select neomycin resistant/transfected cells. G418 was added to the media every 2-3 days for 6-8 weeks. At this point, cells were centrifuged and transferred to Poly-L-Lysine coated 6 well culture plates and stained with crystal violet for visualization of resistant cells. Successful transfection was also determined by analyzing the expression of *green fluorescent protein (GFP)* in the co-transfected *pAluA* + *pLRE3-GFP* containing wells.

## SUPPLEMENTARY MATERIALS FIGURES AND TABLES



## Abbreviations

AA: Anacardic Acid
CT: Cancer Testis
CLA: Cutaneous Lymphocyte-associated Antigen
CTCL: Cutaneous T-Cell Lymphomas
DNA DSBs: DNA double strand breaks
GFP: green fluorescent protein
HAT: histone acetyltransferase
HDAC: histone deacetylase
MF: Mycosis Fungoides
cALCL: primary cutaneous anaplastic large cell lymphoma
PBMCs: Peripheral Blood Mononuclear Cells
REB: Research Ethics Board
SS: Sézary Syndrome
SINE: Short INterspersed Element
