# Supplementary material for: A study of meiomitosis and novel pathways of genomic instability in cutaneous T-cell lymphomas (CTCL)
Source: Oncotarget. 2018 Dec 28;9(102):37647–61. doi: 10.18632/oncotarget.26479 (PMC6340880; doi:10.18632/oncotarget.26479)
Supplement: Supplementary file 1 [file oncotarget-09-37647-s001.pdf]

A study of meiomitosis and novel pathways of genomic instability in cutaneous T-cell lymphomas (CTCL)

SUPPLEMENTARY MATERIALS

Summary of meiosis and detailed description of normal function of the studied genes

The reciprocal exchange of genetic information between homologous chromosomes, or "crossing over" is a defining feature of meiosis (Supplementary Figure 1). In meiotic germ cells, recombination is carried out through the programmed formation and repair of DNA DSBs [1]. DNA DSB creation is an essential step for the initiation of meiosis, and is predominantly mediated by the highly conserved, topoisomerase-like enzyme *SPO11* [1, 2]. Mouse spermatocytes deficient in *SPO11* fail to synapse chromosomes and progress beyond the zygotene stage of meiosis [3]. Upstream of *SPO11, STRA8* is expressed by pre-meiotic germ cells and has been shown to act through retinoic acid signaling, as an essential switch to enter meiosis from mitosis [4, 5] – a decision made during the G1/S checkpoint of the cell cycle [6]. *STRA8* deficient testis and ovarian germ cells are able to undergo normal early mitotic development but fail to undergo premeiotic DNA replication, meiotic chromosome condensation, cohesion, synapsis and recombination [5].

Following the generation of DNA DSBs, *SPO11* is released from the DNA and the 5' ends of the DSB, which are then degraded by endonucleases to yield single stranded DNA tails [2] (Supplementary Figure 1). The meiosis-specific recombinase *DMC1* binds to the single stranded DNA, initiates a search for homology, and catalyzes strand invasion into one of the sister chromatids of the homologous chromosome [7]. Disruption of *DMC1* gene in mice results in arrest of meiosis in germ cells at the early zygotene stage and subsequent apoptosis, due to failure of homologous chromosome synapsis to take place [8]. *HOP2* and *MND1* proteins are also required for the process to occur, and have been shown to form a stable heterodimer that greatly enhances *DMC1*-mediated strand invasion [9]. *RAD51*, which is essential for homologous recombinational repair of DSBs in somatic cells, and is well known to exhibit altered expression in cancer [10], is also required during meiosis where it plays a similar role to but acts independently of *DMC1* [11]. Following strand invasion, resolution of DNA repair results in either a crossover or non-crossover event in meiotic germ cells [12].

In order for recombination and proper chromosomal segregation to occur, DNA must be held together and separated at controlled intervals throughout meiosis by a cohesin complex (Supplementary Figure 1). *REC8* and *STAG3* are two major meiosis-specific subunits of the cohesin complex, and are both required for meiosis [13]. *STAG3* has been shown to be required for the function and stability of all meiosis-specific cohesin complexes [14]. *REC8* is known to play several roles, including kinetochore orientation and adhesion [15]. Namely, *REC8* binds homologous chromosome arms during meiosis I, and the centromere of sister chromatids during meiosis II [16]. *REC8* is degraded from centromeres, when sister chromatids separate during the anaphase of meiosis II, and is normally protected from degradation before this point by *SGO2* [17]. Although *SGO2* is expressed ubiquitously, it is highly expressed in the testis [18] and is required for the progression of meiosis, but not mitosis [19].

In addition, a scaffold-like structure called the synaptonemal complex bridges homologous chromosomes throughout meiosis and is composed of the proteins *SYCP1, SYCP2* and *SYCP3* [20] (Supplementary Figure 1). It is thought that the synaptonemal complex facilitates pairing of homologs and recombination through mechanisms that remain poorly elucidated [21]. Deletion of any of *SYCP1/2/3* results in meiotic arrest and sterility in mice [20, 22, 23]. *HORMAD1* is another critical protein that plays multiple roles during the progression of meiosis. Notably, *HORMAD1* promotes the formation of the synaptonemal complex, is involved in the generation of DSB and also regulates cell cycle checkpoints [24, 25]. It is evident that these genes are tightly regulated throughout the cell cycle and need to be present in the right place and at the right time in the cell in order to form a protein complex and work in concert with other genes to orchestrate this process.

REFERENCES

1. Keeney S, Neale MJ. Initiation of meiotic recombination by formation of DNA double-strand breaks: mechanism and regulation. Biochem Soc Trans. 2006; 34: 523-5. https://doi.org/10.1042/BST0340523.
2. Keeney S. (2008). Spo11 and the formation of DNA double-strand breaks in meiosis. In: Egel R, Lankenau DH, eds. Recombination and Meiosis: Crossing-Over and Disjunction. (Berlin, Heidelberg: Springer Berlin Heidelberg), pp. 81-123.
3. Smirnova NA, Romanienko PJ, Khil PP, Camerini-Otero RD. Gene expression profiles of Spo11-/- mouse testes with spermatocytes arrested in meiotic prophase I. Reproduction. 2006; 132: 67-77. https://doi.org/10.1530/rep.1.00997.
4. Endo T, Romer KA, Anderson EL, Baltus AE, de Rooij DG, Page DC. Periodic retinoic acid–STRA8 signaling

intersects with periodic germ-cell competencies to regulate spermatogenesis. Proceedings of the National Academy of Sciences. 2015; 112: E2347-E56. https://doi.org/10.1073/pnas.1505683112.

5. Anderson EL, Baltus AE, Roepers-Gajadien HL, Hassold TJ, de Rooij DG, van Pelt AMM, Page DC. Stra8 and its inducer, retinoic acid, regulate meiotic initiation in both spermatogenesis and oogenesis in mice. Proceedings of the National Academy of Sciences. 2008; 105: 14976-80. https://doi.org/10.1073/pnas.0807297105.
6. Baltus AE, Menke DB, Hu YC, Goodheart ML, Carpenter AE, de Rooij DG, Page DC. In germ cells of mouse embryonic ovaries, the decision to enter meiosis precedes premeiotic DNA replication. Nat Genet. 2006; 38: 1430-4. http://www.nature.com/ng/journal/v38/n12/suppinfo/ng1919_S1.html.
7. Neale MJ, Keeney S. Clarifying the mechanics of DNA strand exchange in meiotic recombination. Nature. 2006; 442: 153-8.
8. Yoshida K, Kondoh G, Matsuda Y, Habu T, Nishimune Y, Morita T. The mouse RecA-like gene Dmc1 is required for homologous chromosome synapsis during meiosis. Mol Cell. 1998; 1: 707-18.
9. Pezza RJ, Voloshin ON, Vanevski F, Camerini-Otero RD. Hop2/Mnd1 acts on two critical steps in Dmc1-promoted homologous pairing. Genes & Development. 2007; 21: 1758-66. https://doi.org/10.1101/gad.1562907.
10. Thacker J. The RAD51 gene family, genetic instability and cancer. Cancer Lett. 2005; 219: 125-35. https://doi.org/10.1016/j.canlet.2004.08.018.
11. Kurzbauer MT, Uanschou C, Chen D, Schlogelhofer P. The recombinases DMC1 and RAD51 are functionally and spatially separated during meiosis in Arabidopsis. Plant Cell. 2012; 24: 2058-70. https://doi.org/10.1105/tpc.112.098459.
12. Gray S, Cohen PE. Control of meiotic crossovers: from double-strand break formation to designation. Annu Rev Genet. 2016; 50: 175-210. https://doi.org/10.1146/annurev-genet-120215-035111.
13. Garcia-Cruz R, Brieno MA, Roig I, Grossmann M, Velilla E, Pujol A, Cabero L, Pessarrodona A, Barbero JL, Garcia Caldes M. Dynamics of cohesin proteins REC8, STAG3, SMC1 beta and SMC3 are consistent with a role in sister chromatid cohesion during meiosis in human oocytes. Hum Reprod. 2010; 25: 2316-27. https://doi.org/10.1093/humrep/deq180.
14. Hopkins J, Hwang G, Jacob J, Sapp N, Bedigian R, Oka K, Overbeek P, Murray S, Jordan PW. Meiosis-specific cohesin component, Stag3 is essential for maintaining centromere chromatid cohesion, and required for DNA repair and synapsis between homologous chromosomes. PLoS Genet. 2014; 10: e1004413. https://doi.org/10.1371/journal.pgen.1004413.
15. Watanabe Y, Nurse P. Cohesin Rec8 is required for reductional chromosome segregation at meiosis. Nature. 1999; 400: 461-4. https://doi.org/10.1038/22774.
16. Stoop-Myer C, Amon A. Meiosis: Rec8 is the reason for cohesion. Nat Cell Biol. 1999; 1: E125-E7.
17. Kitajima TS, Kawashima SA, Watanabe Y. The conserved kinetochore protein shugoshin protects centromeric cohesion during meiosis. Nature. 2004; 427: 510-7. http://www.nature.com/nature/journal/v427/n6974/suppinfo/nature02312_S1.html.
18. Lee J, Kitajima TS, Tanno Y, Yoshida K, Morita T, Miyano T, Miyake M, Watanabe Y. Unified mode of centromeric protection by shugoshin in mammalian oocytes and somatic cells. Nat Cell Biol. 2008; 10: 42-52. https://doi.org/10.1038/ncb1667.
19. Llano E, Gómez R, Gutiérrez-Caballero C, Herrán Y, Sánchez-Martín M, Vázquez-Quiñones L, Hernández T, de Álava E, Cuadrado A, Barbero JL, Suja JA, Pendás AM. Shugoshin-2 is essential for the completion of meiosis but not for mitotic cell division in mice. Genes & Development. 2008; 22: 2400-13. https://doi.org/10.1101/gad.475308.
20. Yang F, De La Fuente R, Leu NA, Baumann C, McLaughlin KJ, Wang PJ. Mouse SYCP2 is required for synaptonemal complex assembly and chromosomal synapsis during male meiosis. The Journal of Cell Biology. 2006; 173: 497-507. https://doi.org/10.1083/jcb.200603063.
21. Page SL, Hawley RS. The genetics and molecular biology of the synaptonemal complex. Annu Rev Cell Dev Biol. 2004; 20: 525-58. https://doi.org/10.1146/annurev.cellbio.19.111301.155141.
22. Kolas NK, Yuan L, Hoog C, Heng HH, Marcon E, Moens PB. Male mouse meiotic chromosome cores deficient in structural proteins SYCP3 and SYCP2 align by homology but fail to synapse and have possible impaired specificity of chromatin loop attachment. Cytogenet Genome Res. 2004; 105: 182-8. https://doi.org/10.1159/000078188.
23. de Vries FA, de Boer E, van den Bosch M, Baarends WM, Ooms M, Yuan L, Liu JG, van Zeeland AA, Heyting C, Pastink A. Mouse Sycp1 functions in synaptonemal complex assembly, meiotic recombination, and XY body formation. Genes Dev. 2005; 19: 1376-89. https://doi.org/10.1101/gad.329705.
24. Wojtasz L, Daniel K, Roig I, Bolcun-Filas E, Xu H, Boonsanay V, Eckmann CR, Cooke HJ, Jasin M, Keeney S, McKay MJ, Toth A. Mouse HORMAD1 and HORMAD2, two conserved meiotic chromosomal proteins, are depleted from synapsed chromosome axes with the help of TRIP13 AAA-ATPase. PLoS Genet. 2009; 5: e1000702. https://doi.org/10.1371/journal.pgen.1000702.
25. Shin YH, McGuire MM, Rajkovic A. Mouse HORMAD1 is a meiosis i checkpoint protein that modulates DNA double-strand break repair during female meiosis. Biology of Reproduction. 2013; 89: 29. https://doi.org/10.1095/biolreprod.112.106773.

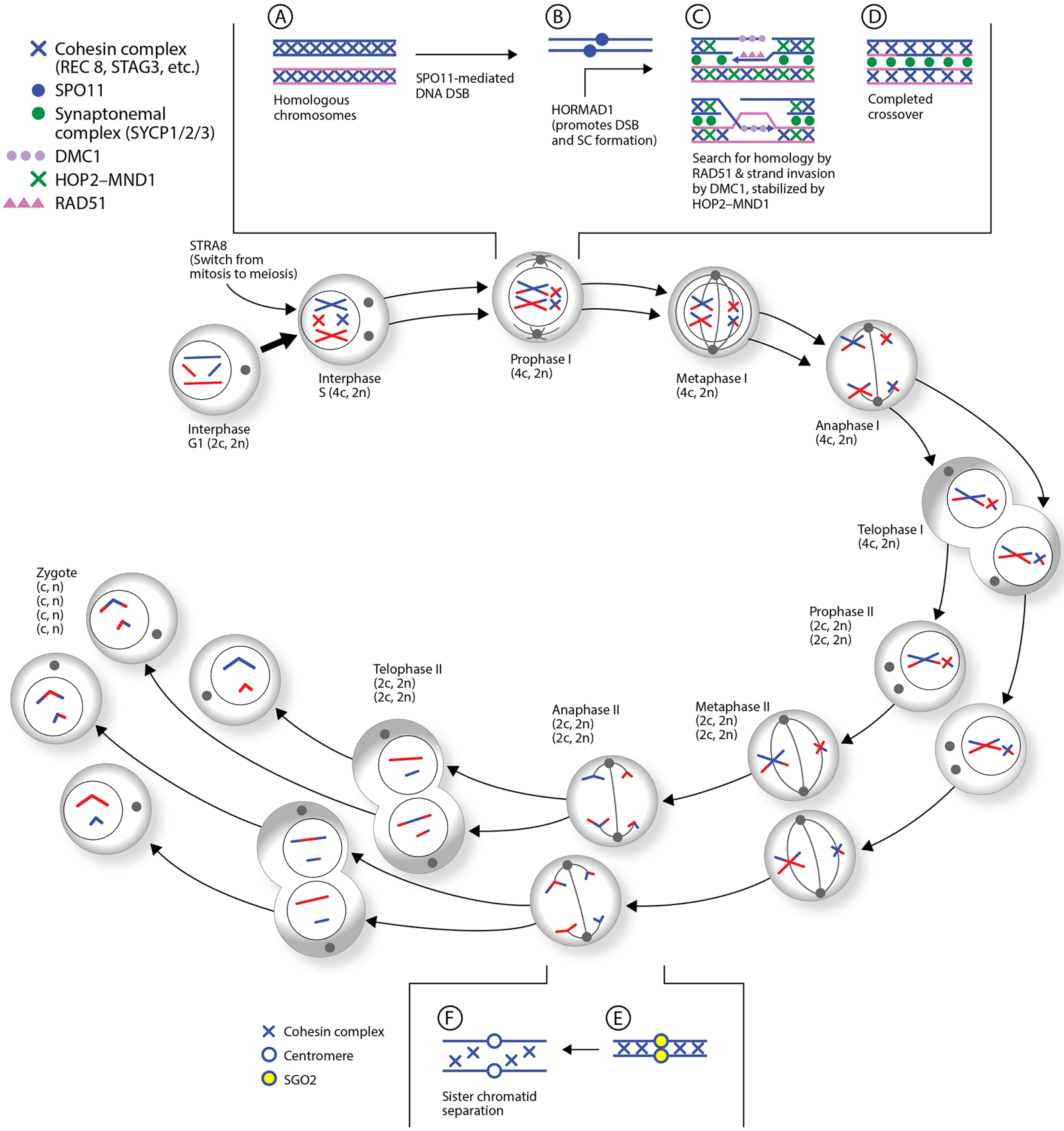

Supplementary Figure 1: Schematic summary of meiosis highlighting the molecular normal functions of *STRA8, SPO11, DMC1, RAD51, MND1, REC8, SGO2, HOMARD1* and *SYCP1* proteins in normal germ cells. *STRA8* acts as an essential switch to initiate meiosis with subsequent recruitment of *SPO11*, topoisomerase-like enzyme that creates DNA DSBs. *DMC1* with the help of *HOP2* and *MND1* binds to single stranded DNA, regulates the search for homology and promotes strand invasion into one of the sister chromatids homologous chromosome. *RAD51* and other proteins execute homologous recombination repair and conclude the crossing over event. During this recombination cohesion complex proteins *REC8* and *STAG3* hold and ensure proper orientation of homologous chromosomes. *SGO2* protects *REC8* from degradation until the beginning of the anaphase. *SYCP1,2* and *3* proteins establish the synaptonemal complex, critical scaffold structure for crossing over to take place. *HOMARD1* promotes the formation of synaptonemal complex and plays additional important roles in regulating meiosis.

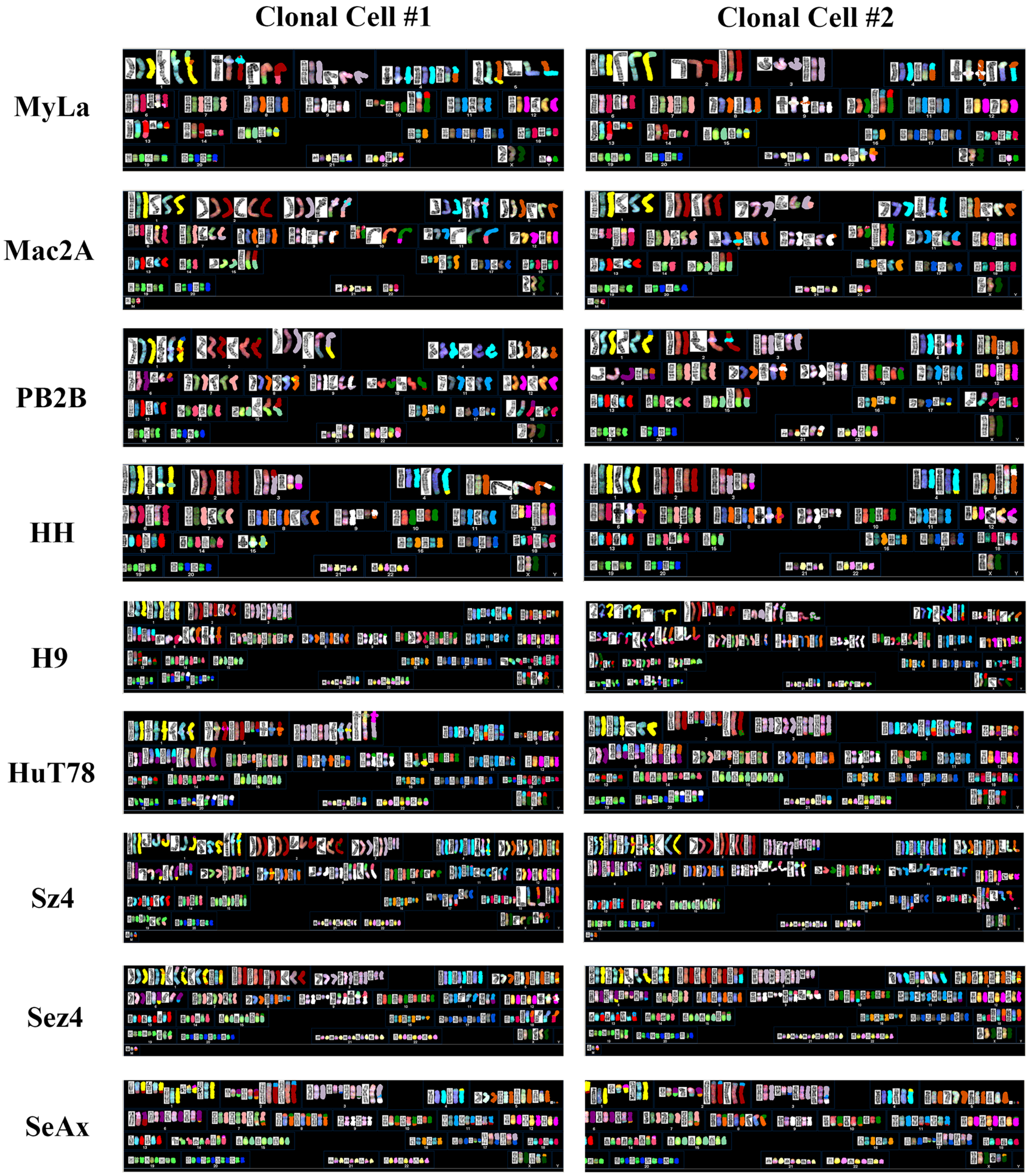

Supplementary Figure 2: G banding and spectral karyotyping of MyLa, Mac2A, PB2B, HH cells representing mycosis fungoides and H9, HuT78, Sz4, Sez4, SeAx cell lines representing Sézary Syndrome. Results show extensive chromosomal abnormalities which varied between clonal cells and between CTCL cell lines.

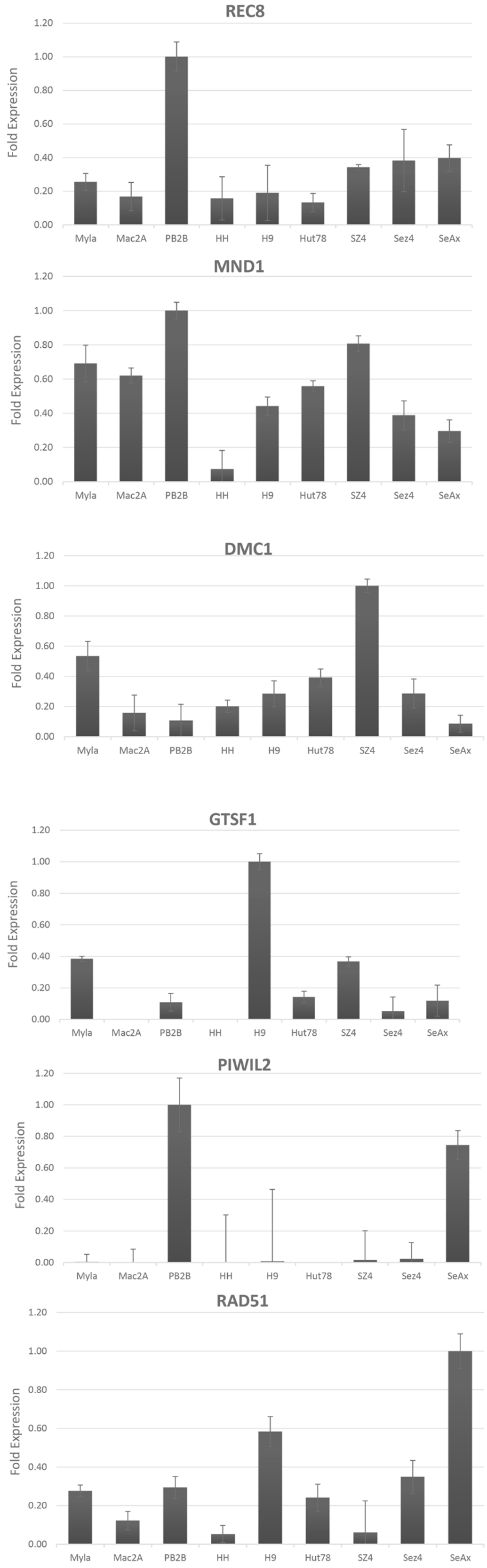

Supplementary Figure 3: RT-PCR expression of meiosis genes in CTCL ce ll lines representative of mycosis fungoides and Sézary Syndrome. For every gene analyzed the highest expression value in our samples was set as 1-fold of expression. (*Continued*)

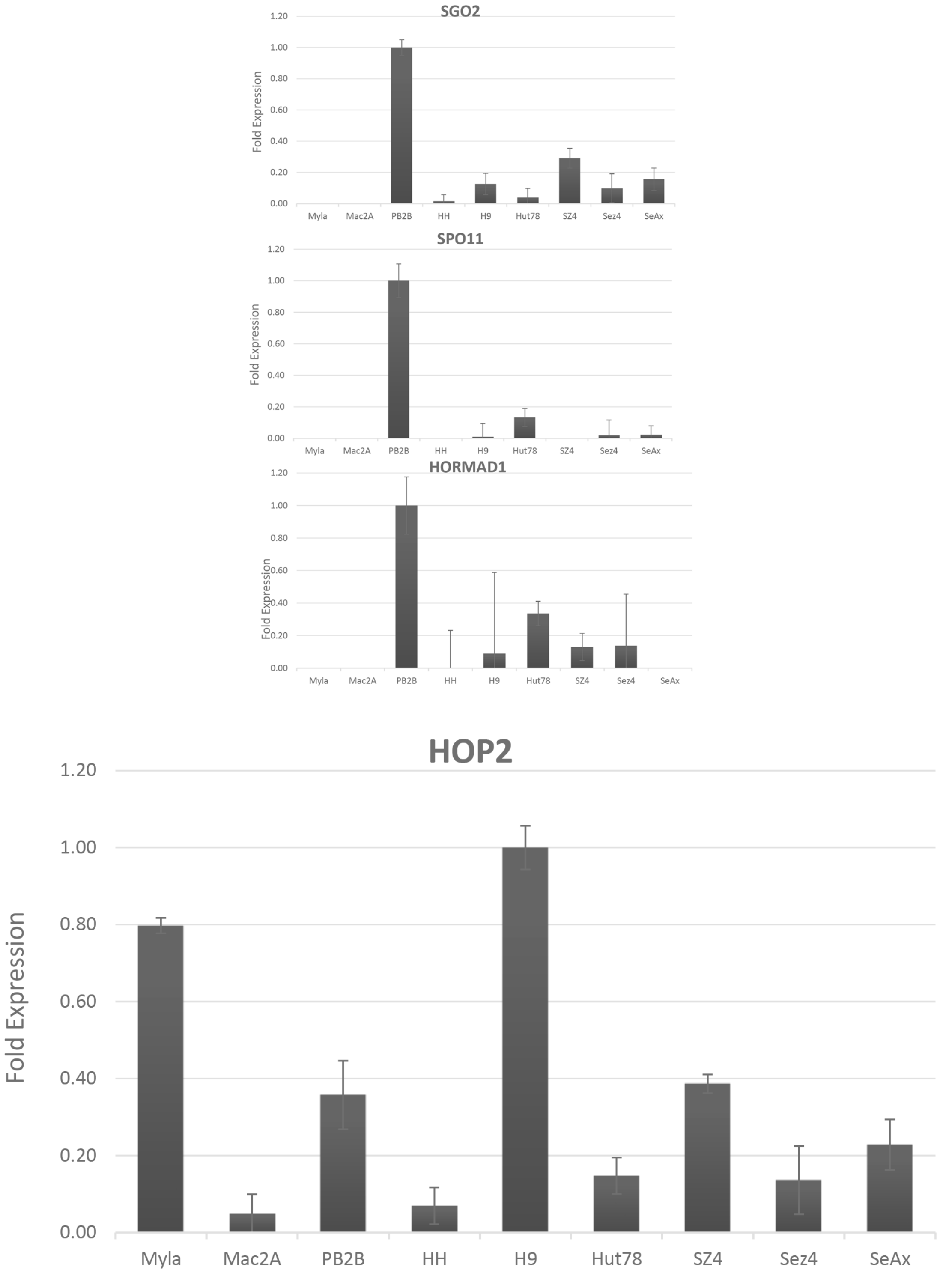

Supplementary Figure 3: (*Continued*) RT-PCR expression of meiosis genes in CTCL ce ll lines representative of mycosis fungoides and Sézary Syndrome. For every gene analyzed the highest expression value in our samples was set as 1-fold of expression.

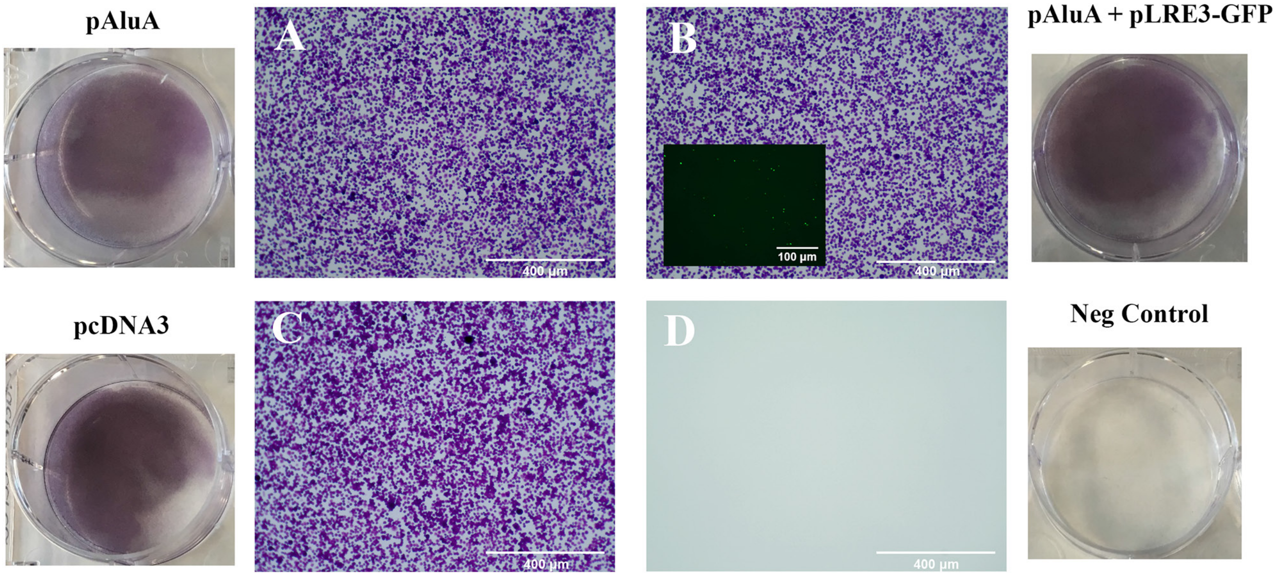

Supplementary Figure 4: LINE-1 machinery is functionally active in H9 cells. A previously described *in vivo* assay [42] was performed to test the ability of endogenously expressed *LINE-1* to provide the necessary protein machinery for Alu retrotransposition to occur. **(A)** H9 lymphocytes were transfected by nucleofection with *pAluA* "reporter" plasmid containing an *Alu* element and a modified *mneoI* cassette that allows the detection of a retrotransposed RNA polymerase III transcribed RNA. **(B)** H9 cells were transfected with *pAluA* "reporter" plasmid plus "driver" retrotransposition-competent human *LINE-1* element *LRE3* tagged with *GFP* (inlay) to monitor transfection efficiency. **(C)** H9 cells were transfected with empty vector control plasmid with the *CMV* promoter and a neomycin-resistance marker or **(D)** Negative control: plasmid not coding for G418 resistance. Transfected cells were grown in suspension and treated with G418 for 6-8 weeks to select for antibiotic resistance conferred in by retrotransposition events (A and B). Cells were subsequently fixed and stained with crystal violet. Images of entire wells (outer panels) and brightfield microscopy are shown (Scale bar = 400 µm).

Supplementary Table 1: Clinical characteristics of patients, who provided blood samples for research

Age & sex	Skin Color	Skin involvement	Blood involvement/ TCR Clonality analysis	Treatments	Diagnosis (Clinical Stage)
75 M	Caucasian	80% body surface area involvement with erythematous scaly plaques on the body	Sézary count: 80% Flow cytometry: 85% T-cells; 80% $CD7^-$/ $CD26^-$ CD4:CD8 ratio >10 Blood: TCR ß clonality	Topical Steroids Isotretinoin, Interferon α, ECP, Brentuximab vedotin	Sézary Syndrome (Clinical Stage IVA_2)
74M	Caucasian	85% body surface area involvement with exfoliative erythroderma	Sézary count: 68% Flow cytometry: Atypical T- cells with discrete loss of CD26 and CD7. CD4:CD8 ratio: 12 Blood: TCR ß, δ and γ clonality	Interferon α, Alitretinoin, NBUVB	Sézary Syndrome (Clinical Stage IVA_2)
72 F	Caucasian	80% body surface area involvement with erythematous patch lesions	Sézary count: 38% Flow cytometry: Abnormal T- cells with discrete loss of $CD26^-$ in 50% of cells. CD4:CD8 ratio: 21 Blood: TCR ß, δ and γ clonality	Topical Steroids NBUVB, Alitretinoin Interferon α,	Sézary Syndrome (Clinical Stage IVA_2)

Supplementary Table 2: Primer sequences used for RT-PCR analyses

Gene Name	Forward Sequence	Reverse Sequence
DMC1	TGCAATGTCAAAGGACTCTCAGAAGC	CCCGGTGGTGATATGGAAAACCA
GTSF1	GCAGACCAGCACCCCATTTGTC	GGCAGAGATTTGGGAACTCGCA
HOP2	CCTCCCCTGCCTTTCTCTCCG	CTCCAGCGTCTTCACCACCAC
HORMAD1	ATCCTAATGCAAAATCTGGGGCCTT	CGGGAGGCTGGTAATCTGGGG
MND1	CTTGGAGAAGATTGCTCCCAAAGAGA	TCCTCTCACAGTCAACCATACCATCA
PIWIL2	GCTGGGGACAGCAAGATGGC	TGTGCTGAAGGTACAGGGAGGC
RAD51	TTTGGCCCACAACCCATTTCAC	CCTCCACAGTATGGAATCCAGCTT
REC8	TGATGGAGACCCTAGAAGATGCTCC	ACTCTCTCTGGGATTGCAGCCT
SGO2	TGGAAACTGGCTCACTTTTTACCTCA	AGCTAATGCCCTGTTGTTGTGCT
SPO11	ACAGAGCAACACTTATGCAACCAAAAG	ACTCCTCCTTGACACTTTTAACATGCA
